# Gender differences in long term sickness absence

**DOI:** 10.1186/s12889-024-17679-8

**Published:** 2024-01-15

**Authors:** Sheila Timp, Nicky van Foreest, Corné Roelen

**Affiliations:** 1 Arbo Unie, Laan Corpus Den Hoorn 102, 4, Groningen, 9728 JR the Netherlands; 2 Faculty of Economics and Business, University of Groningen, Nettelbosje 2, Groningen, 9747 AE the Netherlands

**Keywords:** Sickness absence duration, Gender, Long term sickness absence, Mental, Musculoskeletal

## Abstract

**Purpose:**

Sickness absence is a major public health problem, given its high cost and negative impact on employee well-being. Understanding sickness absence duration and recovery rates among different groups is useful to develop effective strategies for enhancing recovery and reducing costs related to sickness absence.

**Methods:**

Our study analyzed data from a large occupational health service, including over 5 million sick-listed employees from 2010 to 2020, out of which almost 600,000 cases were diagnosed by an occupational health physician. We classified each case according to diagnosis and gender, and performed descriptive statistical analysis for each category. In addition, we used survival analysis to determine recovery rates for each group.

**Results:**

Mean sickness duration and recovery rate both differ significantly among groups. Mental and musculoskeletal disorders had the longest absence duration. Recovery rates differed especially during the first months of sickness absence. For men the recovery rate was nearly constant during the first 1.5 year, for women the recovery rate was relatively low in the first three months, and then stayed nearly constant for 1.5 year.

**Conclusion:**

Across almost all diagnostic classes, it was consistently observed that women had longer average sickness absence durations than to men. Considering mental disorders and diseases of the musculoskeletal system, women had relatively lower recovery rates during the initial months compared to men. As time progressed, the recovery rates of both genders converged and became more similar.

## Introduction

The average sickness absence rates vary between 3% and 6% across European countries. Especially long term sickness absence (LTSA) has high costs amounting up to 2.5% of a country’s gross domestic product [1]. In the Netherlands, most costs are carried by employers, as they not only lose the productivity of the sick-listed employee but also have to continue to pay the salary of sick employees for two years [2]. Reducing sickness absence is important for society, the employer, and in particular for employees, as being in employment is often associated with better quality of life, health and physical functioning [3].

Sickness absence is related to various factors, including the cause of the sickness absence, the age and gender of the employee, and the work environment. The most common causes of LTSA are diseases of the circulatory system, mental disorders and diseases of the musculoskeletal system [4, 5]. In the Netherlands, the sickness absence percentage increases with age until 65 years of age. Interestingly, employees between the ages of 65 and 75 exhibit a lower sickness absence percentage than those aged 55 to 64 years, suggesting a “healthy worker” effect. This phenomenon implies that healthier individuals tend to remain in the workforce longer, while those with health issues exit the labor process at an earlier stage [4].

Extensive research has been done to investigate gender differences in sickness absence. The predominant finding of these studies is that, on average, female employees report sick more frequently and experience longer periods of sickness absence in comparison to male employees [6–11]. Leijon et al. (1998) investigated gender trends in sickness absence for various causes, and found that women had both a higher sickness absence frequency and longer sickness absence duration compared to men [8]. Similarly, Arcas et al. (2016) found that among employees with diseases of the musculoskeletal, women had a longer sickness absence duration than men [9]. However, in some older age groups, they observed a longer absence duration for men. Labriola et al (2011) focused on long term sickness absence and found that the frequency was nearly 40% higher for men compared to women [10].

Bekker et al. (2009) conducted a literature review on the relationship between gender and sickness absence, finding that women are generally absent more frequently, especially when it comes to short-term absences [12]. They also found that gender differences in sickness absence are influenced by various factors such as country of residence, age, and professional group. Different other studies focus on factors explaining the so called gender-duration-gap, such as parenthood, type of work, and social roles [13–17]. Nilsen et al. (2017) reviewed eight longitudinal studies and found that although women report higher work-family conflict than men, but this did not explain the gender difference in sickness absence [17]. Angelov et al. (2013) investigated the effect of parenthood on sickness absence and found that entering parenthood increased women’s absence rate compared to the corresponding rate for men. They also found that this effect was long-lasting and remained at least until 16 years after the birth of first child [13]. On the other hand, Mastekaasa (2013) analyzed data from 23 EU countries plus Norway and found that dependent children are associated with lower sickness absence among married/cohabiting women [16]. Casini et al. (2013) also studied factors that could explain gender differences in absence duration. They found that especially job strain is linked to a longer absence duration for women compared to men [14]. Similarly, Lidwall et al. (2009) found that women have a higher risk on long-term absence when working in high-strain jobs compared with men, especially in the private sector [7].

Contrarily, some other studies have found limited evidence of a correlation between sickness duration and gender. For example, Cornelius et al. (2010) conducted a systematic review and found only limited evidence to support an association between sickness absence duration and gender [18]. A study by Spierdijk et al. (2009) on self-employed individuals failed to identify any significant gender differences in sickness absence duration [19].

While most studies have primarily focused on statistical measures such as average duration of sickness absence or sickness absence frequency, our study takes a more comprehensive approach. In addition to determining descriptive statistical measures, we investigate gender differences by analyzing recovery rates across various diagnoses. This thorough analysis provides us with a more detailed understanding of how gender influences sickness absence and the trajectory of recovery across various diagnoses over time.

## Methods

### Study population and design

In The Netherlands, it is a requirement for all employers to ensure that their employees have access to occupational health care, which is typically provided by an occupational health service (OHS). An OHS is responsible for registering sickness absences, and for providing guidance to sick-listed employees by medical consultations and advice for returning to work (RTW). When an employee reports sick, the OHS registers this in the sickness absence register. Sickness absence can be due to any (i.e., work-related and non-work-related) physical or mental illness or injury. In The Netherlands, the employer financially compensates sickness absence for a period of 104 weeks. Most employers cover 100% of the worker’s salary in the first year of sickness absence and 70% in the second year. The OHS follows employees during 104 weeks of sickness absence, after which the employee may apply for a disability pension provided by the Employee Insurance Agency (UWV) and the employer may end the job contract.

For our study we retrieved data from a sickness absence register of a large Dutch national OHS, registering sickness absence data of approximately 1.24 million Dutch employees from about 11.6 thousand companies of various economic sectors throughout the country. The dataset included all reported employee sickness cases from January 2010 to December 2020. When an employee experienced multiple periods of sickness absence, we included each separate period in the dataset as a single case. For each case, we calculated the duration of sickness, defined as the interval from the first to the last registered day of sickness absence. For our study we included cases aged between 16 and 70 with a sickness duration between 1 day and 104 weeks. We excluded cases diagnosed as pregnancy and pregnancy-related diseases.

Most of the sickness absence cases we observed were short-term, typically lasting less than two weeks. These short-term absences were commonly due to medical conditions like upper respiratory infections or gastrointestinal disturbances. For longer sickness absence periods, employees consult an occupational health physician (OHP) for return-to-work (RTW) advice. In the Netherlands, it is mandatory for employees on sick leave to consult an OHP within 42 days of their absence. The OHP then documents the diagnosis in the Occupational Health Service (OHS) register. To classify the employees’ diagnoses, the OHS uses the Dutch classification system for Occupational and Social insurance physicians (CAS) [20]. This system is based on the ‘International Statistical Classification of Diseases and Related Health Problems’ (ICD-10) and contains similar main categories. An important distinction between the CAS system and the ICD10 is the classification of neoplasms. In the CAS system, neoplasms are categorized under the relevant organ system, whereas in the ICD10, they are considered a distinct class [21].

For our detailed analysis, we utilized cases that had been OHP diagnosed, excluding cases with unknown diagnoses.

### Analysis

Statistical analyses were done using the *lifelines* library for survival analysis and Python 3.10 [22]. Employees were right-censored when the job contract ended during the sickness absence. Data about sickness duration were analyzed descriptively using the mean, median, and standard deviation.

Descriptive statistics were computed for each diagnostic category and gender. For each diagnostic category, the difference in means between genders was calculated. We focused on differences in means including confidence intervals instead of applying tests of significance. This is because the latter are heavily influenced by sample size and will almost always demonstrate a significant difference, even for small differences that may not have practical significance [23].

We have analyzed the most important causes for sickness absence, in terms of both frequency and duration, in more detail. For these diagnostic categories, hazard rates have been determined using the Nelson-Aalen estimator [24]. The estimated hazard function gives the recovery rate at each point in time *t*, and is defined as the probability that an employee will recover in the next moment $t + 1$, conditional on the fact that the employee was still sick at time *t*. For instance, if 100 employees are absent at the start of a day, and 2 have recovered by the start of the next day, the daily recovery rate is 0.02.

Considering that the onset and recovery of sickness absence are not evenly distributed across weekdays, with a higher percentage of employees reporting recovery on Mondays, we used a one-week moving average filter to smooth hazard the rate $h_t$ at each point in time *t*.

1 $$\begin{aligned} h_t = \frac{1}{n} \sum \limits _{i=-3}^{i=3}h_{t+i}, \text { where } n = 7 \end{aligned}$$

**Ethical approval** Ethical approval was not necessary as the Medical Research involving Human Subjects Act does not apply to studies of anonymized register data. The Medical Ethics Committee of the University Groningen confirmed that ethical clearance was not necessary for this study.

## Results

### Descriptive results

**Fig. 1:**
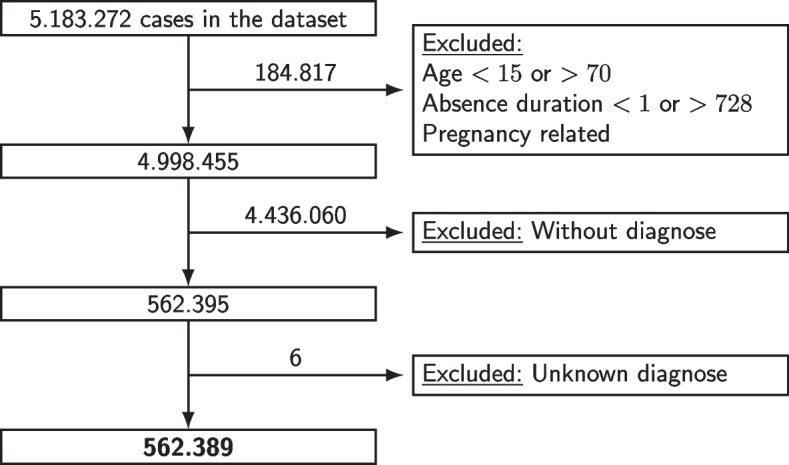
Flowchart of the inclusion and exclusion of cases used for the analysis

In the period between January 2010 and December 2020 there were 4,998,455 sickness cases that fulfilled our inclusion criteria, cf., Fig. 1. Of these cases 52% were male, and 48% were female. This closely reflects the male/female ratio of the Dutch working population during the same period (about 53% male and 47% female) [25]. The mean absence duration was 23 ± 42 days. Among these approximately 5 million cases, approximately 11% (*n* = 562,395) were consulted and diagnosed by an occupational health physician (OHP). For further analysis, we excluded cases where employees were diagnosed with an unknown code (*n* = 6).

Tables 1 and 2 present descriptive statistics regarding sickness absence duration for men and women across various diagnostic categories. The most prevalent causes of sickness absence were diseases of the musculoskeletal system, mental disorders, diseases of the respiratory system, and diseases of the digestive system. For women, the most prevalent diagnoses were mental disorders, whereas for men diseases of the musculoskeletal system were most prevalent. Across all OHP diagnosed cases, the mean sickness absence duration was 158 days for women and 117 days for men, with an average gender difference of 41 days (95% confidence interval: 40.3-42.0). Among the most prevalent causes, mental disorders had the longest sickness absence duration (186 ± 162 days).

Except for diagnoses of diseases of the blood and blood forming organs, women had a longer average sickness absence duration than men. For each specific diagnosis, the 95% confidence interval indicates that the results are not only statistically significant but also practically relevant. The largest gender differences in sickness absence duration were found for cases diagnosed with diseases of the musculoskeletal system and cases diagnosed with diseases of the genitourinary system.

**Table 1:** General statistics for different causes of sickness absence. Duration is the mean sickness absence duration ± the standard deviation, $\#$ gives the number of male and female cases respectively

Diagnosis	ICD10	nr cases	duration	age	$\#$ female	$\#$ male
All cases with diagnosis		562389	135 ± 155	46	243385	319004
Diseases of the musculoskeletal system	M00-M99	172892	128 ± 141	47	64417	108475
Mental and behavioral disorders	F00-F99	158041	186 ± 162	44	87788	70253
Diseases of the respiratory system	J00-J99	68070	47 ± 103	46	23904	44166
Diseases of the digestive system	K00-K93	36000	90 ± 138	46	13648	22352
Symptoms/signs not elsewhere classified	R00-R99	35045	74 ± 127	43	13942	21103
Diseases of the nervous system	G00-G99	23569	171 ± 187	46	11264	12305
Diseases of the circulatory system	I00-I99	24919	186 ± 166	53	5768	19151
Diseases of the genitourinary system	N00-N99	20001	173 ± 176	49	13135	6866
Diseases of the skin/subcutaneous tissue	L00-L99	7504	90 ± 122	47	2549	4955
Diseases of the eye and adnexa	H00-H59	5389	113 ± 144	50	1851	3538
Endocrine diseases	E00-E90	4722	176 ± 167	48	2571	2151
Diseases of the ear and mastoid process	H60-H95	3940	118 ± 149	48	1584	2356
Diseases of the blood/blood-forming organs	D50-D89	2297	249 ± 211	48	964	1333

**Table 2:** Gender statistics for different causes of sickness absence. Duration is the mean sickness absence duration ± standard deviation. $\Delta$ duration gives the gender difference in mean sickness duration and the corresponding 95% confidence interval

Diagnosis	duration ♀	duration ♂	$\Delta$ duration (95% CI)
All cases with diagnosis	158 ± 162	117 ± 147	41 (40.3 - 42.0)
Diseases of the musculoskeletal system	154 ± 151	113 ± 132	41 (39.8 - 42.7)
Mental and behavioral disorders	197 ± 163	173 ± 159	24 (22.3 - 25.5)
Diseases of the respiratory system	58 ± 114	41 ± 96	17 (15.2 - 18.6)
Diseases of the digestive system	104 ± 141	82 ± 136	23 (19.7 - 25.6)
Symptoms/signs not elsewhere classified	100 ± 141	57 ± 113	43 (40.1 - 45.7)
Diseases of the nervous system	178 ± 188	165 ± 186	13 (8.0 - 17.5)
Diseases of the circulatory system	195 ± 174	183 ± 164	12 (7.4 - 17.5)
Diseases of the genitourinary system	191 ± 183	137 ± 156	54 (49.4 - 59.1)
Diseases of the skin/subcutaneous tissue	114 ± 133	78 ± 114	36 (30.3 - 42.4)
Diseases of the eye and adnexa	132 ± 155	102 ± 137	30 (21.9 - 38.6)
Endocrine diseases	192 ± 166	156 ± 165	36 (26.2 - 45.2)
Diseases of the ear and mastoid process	138 ± 156	105 ± 143	33 (23.3 - 42.6)
Diseases of the blood/blood-forming organs	231 ± 204	262 ± 216	-31 (-48.6 - -14.0)

### Analytical results

The most important causes for sickness absence, in terms of both frequency and duration, were diseases of the musculoskeletal system, mental disorders, diseases of the nervous system and diseases of the circulatory system. We have explored these diagnostic categories in more detail.

**Fig. 2:**
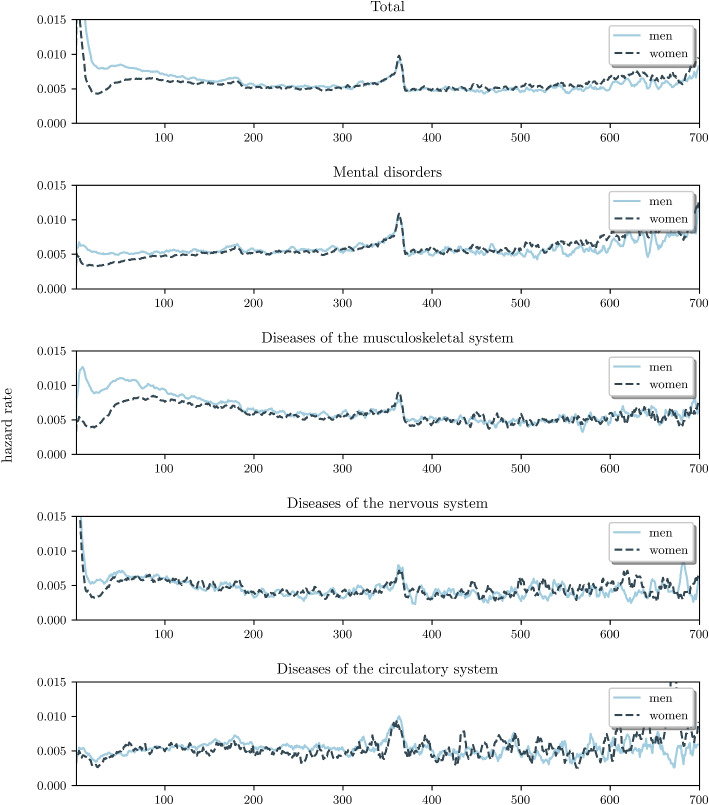
Plots of hazard (recovery) rate as a function of time (in days) for the most common causes of long term sickness absence. In particular in the first few months of absence, the hazard rates for women are lower than those for men. This difference is the most pronounced for mental disorders and diseases of the musculoskeletal system. For diseases of the circulatory system hazard rates are similar for men and women

Figure 2 displays the recovery rates for these diagnostic categories. For male employees with mental disorders, the recovery rate remains rather constant during the initial 1.5 years. This suggests that the conditional probability of recovery does not change during this period and is independent of the actual sickness absence duration. In contrast, for female employees with mental disorders, we observe considerably lower recovery rates in the first few months. This indicates that a smaller proportion of women recover during this early period relative to men.

For diseases of the musculoskeletal system, the gender difference in recovery rate is even more pronounced, particularly in the initial few months, where the percentage of women reporting recovery is relatively low. However, after approximately three months, the recovery rates become comparable between women and men. For diseases of the nervous system we observe a small difference in recovery rate during in the initial stage of sickness absence. The recovery rates for diseases of the circulatory system are similar for men and women.

Table 3 shows the proportion of cases with either mental or musculoskeletal disorders that have recovered at three different points in time (13, 26, and 52 weeks). We see that the proportion of recovered cases differs most among genders within the first three months. For instance, after 3 months, 67% of men with musculoskeletal disorders have recovered, versus 51% of women. For mental disorders, the proportions are 44% for men and 37% for women.

**Table 3:** Fraction of male and female cases that have recovered at different points in time (in weeks) for mental disorders and disorders of the musculoskeletal system

	Mental		Musculoskeletal	
time (weeks)	men	women	men	women
13	0.44	0.37	0.67	0.51
26	0.69	0.62	0.85	0.76
52	0.9	0.88	0.92	0.95

Besides gender differences the recovery curves show another interesting pattern around one year of sickness absence. Around that period, we observe a sudden increase in recovery rate for both men and women. This might be an effect of a decrease in salary in the second year of sickness absence, as most employers decrease the salary to 70% after the first year of sickness absence. We will investigate this topic in more detail in a future paper.

## Discussion

We studied sickness absence patterns among men and women for different diagnostic categories. The results show that for almost all categories the average sickness absence duration was remarkably longer for women than for men. The largest gender differences in sickness absence duration were found for cases with musculoskeletal disorders and diseases of the genitourinary system. The large difference in absence duration for diseases of the genito-urinary system can be explained as this category includes diseases of the breasts, such as mamma carcinoma, which are more prevalent among women and in general are associated with a long sickness absence duration.

The most common causes for sickness absence were diseases of the musculoskeletal system, mental disorders, diseases of the nervous system and diseases of the circulatory system. For these categories gender differences were analyzed in more detail by determining the recovery rate over time.

In what follows, we will compare our current results with prior studies. To the best of our knowledge, there are only some Dutch studies exploring recovery rates in depth, which might be due to the fact that Dutch regulations for LTSA are rather generous as compared to other countries. However, given that gender differences exist in many Western countries, the observed differences in recovery rates during the initial months might be observed in other countries as well.

The general opinion is that the probability of recovery decreases with increasing sickness duration, or in other words, a negative duration dependency [6, 26], although some studies report a positive duration dependence [27]. Koopmans et al. (2009) studied long-term sickness absence between 1998 and 2001 and found declining recovery rates over time [26]. In contrast, our study found relatively stable recovery rates during the entire duration of sickness absence, except for the initial months. One important difference between our study and Koopmans et all’s is that we conducted subgroup analyses by gender and type of disease. Nonetheless, given that recovery rates were relatively stable across all diagnoses and genders, we anticipate a similarly stable combined recovery rate. Another key difference is that sickness policies in the Netherlands have changed over time. Before 2004, employers were financially responsible for sick employees for only one year, after which individuals could apply for a disability pension. Under the current regulation employers are financially responsible during the first two years of sickness absence. This extended period of financially responsibility could potentially motivate employers to more actively support employee recovery and thus possibly influence recovery rates. Conversely, the prospect of a disability pension under the previous policy might theoretically also have had a stimulating effect on the recovery rate.

Joling et al. (2006) examined the duration dependence during sickness absence and found that the recovery rate increased over time [27]. Their study analyzed both short-term and long-term sickness absence for employees who reported sick in 1990. In contrast, our study only focused on long-term sickness absence and included more recent cases. Roelen et al. (2012) investigated the recovery rates for employees that had been sick-listed between 2006 and 2008 with mental disorders and also found that women resumed their work later than men [6]. This finding is consistent with our results for mental disorders.

By examining recovery rates for different diagnostic groups over the entire duration of sickness absence, our study additionally found that not only the average sickness duration differs between men and women, but that there are also remarkable differences in the recovery rates. In particular during the first months of sickness absence, women have a lower recovery rate than men, indicating women have a certain “delay” in recovery. In the next paragraph, we will explore possible reasons for these observed differences, and discuss their consequences in a practical context.

### Gender-related factors

Various gender-related factors may contribute to differences observed in absence duration and recovery rate, including medical, biological, personal, family and work-related factors [12]. We will investigate some of these factors and explore how these could account for the observed delay in recovery for women compared to men.

**Person-related factors** Person-related factors, such as coping style and work attitude, can also play a role in sickness absence. Tamres et al. (2002) conducted a meta-analysis of 50 studies on gender differences in coping and identified 17 coping strategies which they classified as problem-focused or emotion-focused behaviors [28]. Problem-focused coping strategies include active behaviors (such as changing the situation, removing the stressor), planning (review possible solutions), seeking instrumental social support that is directed towards solving problems, and general problem-focused behavior. Emotion-focused behaviors aim to alter the response to the stressor and include seeking emotional support, avoidance, denial, positive reappraisal, isolation, venting, rumination, wishful thinking, self-blame, positive self-talk, and exercise. According to the study, women tend to make more use of coping strategies compared to men, particularly more emotion-focused strategies. Van Rhenen et al. (2008) investigated the role of different coping styles on both the duration and frequency of sickness absence [29]. They found that both the use of an active problem-solving coping style and seeking emotional support decreased the mean sickness absence duration, with a stronger effect of the problem-solving coping style. Other emotion-based strategies had either no effect (expression of emotions) or a negative effect (avoidance) on sickness absence duration. Conversely, Loset et al. (2018) conducted a survey study to explore differences in attitudes and norms regarding sickness absence and found no significant differences between genders [30].

Further research is necessary to investigate variations in coping styles throughout the entire period of sickness absence, with particular emphasis on potential differences in coping styles during the initial stage of sickness absence and on changes in coping styles after these initial period.

**Daily life characteristics ** Several differences in daily life and occupational characteristics may also influence sickness absence frequency and duration [13, 15–17]. Women generally tend to spend more time to household tasks and childcare compared to men. The double burden hypothesis proposes that the combination of different roles, such as being an employee and a parent, can increase stress and consequently increase the risk for sickness absence. The strain associated with having multiple roles can be reflected by perceived work-family conflicts, where the demands of one’s professional role interfere with their family role, or vice versa. In a systematic review by Nilsen et al. (2017), they found moderate evidence for a positive correlation between work-family conflict and subsequent sickness absence, indicating that the strain from balancing work and family roles can indeed lead to higher levels of sickness absence [17]. However, the evidence was insufficient to draw conclusions about the role of gender in the prospective association between work-family conflict and subsequent sickness absence.

In our study we found that gender differences in particular influence the recovery rate during the first months of sickness absence. The work-family conflict provides a possible explanation for this observed differential recovery. The strain associated with managing different roles may affect the recovery process differently for men and women. For instance, during the initial recovery phase, women might prioritize resuming their family and social roles, while men may focus more on returning to their professional roles. This potential variance in prioritization could account for the lower recovery rates among women during the early stages of sickness absence. Further research is necessary to investigate the relationship between work-family conflict and recovery rates during the complete sickness absence period.

**Occupational characteristics** In addition to daily life characteristics, sector-specific gender representation patterns may also play a role. Women are predominantly employed in the healthcare, social services, and education sectors, whereas men are more commonly found in industries such as construction, manufacturing, information technology, and transportation [31]. Some studies suggest that higher rates of sickness absence are associated with occupations dominated by women [32, 33]. The emotionally demanding nature of jobs in healthcare and social services often entails working directly with patients or clients. Such roles may require a more complete recovery from mental disorders before work can be resumed. In contrast, physically demanding work might offer a distraction from mental health issues, which could partly explain why men might return to work sooner.

However, studies about the association between job occupation and sickness absence are not conclusive. In a recent study Østby et al. (2018) found no evidence that the type of occupation is related to gender differences in sickness absence [15]. Mastekaasa (2014) even found an increase in gender differences when adjusting for the type of occupation [11].

**Convergence of recovery rates** Interestingly, recovery rates for men and women become more similar after the initial three months of sickness absence. This could be due to the natural course of the disease, a change in coping techniques or a re-evaluation of work. More research is needed to investigate the reasons behind this change in recovery rates, which could have significant implications for sickness absence management strategies.

**Strengths and Weaknesses of the Study** The strength of our study is that we could analyze a very large sample of sickness cases over a period of 10 years. Furthermore, we have used the occupational health physician’s (OP) diagnosis, enabling us to investigate sickness absences across various diagnostic categories. A limitation of our study is that we did not examine the impact of other factors that could potentially influence sickness duration and recovery rates. These factors include variations in job type, age, socio-economic status, specific diagnoses, overall health status, and the severity of the disorder. To improve understanding of gender differences in sickness absence, further research is needed to investigate the effect of these factors in particular during the first months of sickness absence.

## Conclusions

Our study found marked gender differences in both sickness absence duration and recovery rates, with a longer sickness absence duration for women compared to men across almost all sickness causes. Interestingly, we found that recovery rates for women were considerably lower in the first months, indicating that most women start later with recovery than men. This indicates that there is a kind of delay in the recovery process for women. However, after the initial months, recovery rates for both genders tend to converge.

In the initial months of sickness duration, a considerable number of employees are affected, so even small differences during this period can greatly influence the total sickness duration and the corresponding costs. Consequently, it is very important to comprehend the factors leading to the noticeable delay in recovery for women. There is a need to further explore known factors that may affect the duration of sickness absence, such as coping mechanisms and conflicts between work and family life. Future research might be directed towards understanding how these factors change during the early stages of sickness absence, as well as the differences among genders in these factors during the beginning phase of sickness absence. This understanding can help to develop effective prevention and intervention strategies to minimize recovery delays and reduce the overall period of sickness absence. Ideally, implementing these strategies during the initial stage of sickness absence seems to be most beneficial.

## Data Availability

The data that support the findings of this study are available from the authors upon reasonable request. The contact person for requests is Sheila Timp and can be contacted by email sheila.timp@arbounie.nl.
